# How can psychiatrists make mental health services more accessible for people with autism?

**DOI:** 10.1192/bjo.2021.1036

**Published:** 2021-10-28

**Authors:** Ashok Roy

**Affiliations:** Psychiatry of Intellectual Disability, Coventry and Warwickshire Partnership NHS Trust, Brooklands Hospital, UK.

**Keywords:** Autism services, reasonable adjustments, workforce competencies, role of psychiatrists, improved outcomes

## Abstract

Unrecognised psychiatric and medical conditions can lead poorer health outcomes, lower health-related quality of life and increased mortality in people with autism, compared with the general population. A reasonable adjustment required in mainstream services is patient prioritisation by clinicians with knowledge and understanding of autism. Developed as part of the revised autism strategy, the recently developed autism competency framework lists the range of capabilities that psychiatrists who treat people with autism should have. Psychiatrists could lead the workforce transformation required to make the reasonable adjustments to mainstream mental health services needed to improve outcomes for people with autism.

Autism is a heterogeneous neurodevelopmental condition characterised by impairments in social communication and interaction, the presence of patterns of restrictive and repetitive behaviours, and focal interests that may include unusual sensory awareness of their environment. Individuals along the spectrum exhibit a full range of intellectual functioning and language abilities.^1^ Comorbid disorder, notably emotional disorder, is frequent and, if unrecognised, can be misdiagnosed as schizophrenia or personality disorders.^2^

In a case–control prevalence cohort study of the total population sample of all English children, adolescents and young adults aged 2–21 years in state-funded education, Roman-Urrestarazu^3^ et al demonstrated an increasing prevalence overall compared with 10 years previous. They also found highest prevalence in Black pupils. The standardised prevalence of autism spread across England was varied. The authors suggested possible reasons such as inconsistent diagnostic processes, variability in the provision of education and special educational support across England, different thresholds for accessing special educational needs and disability support or an education and health care plan, differences in prevalence between different areas and large variance in male/female ratios. In a population-based study, Rai et al^4^ found that lower socioeconomic status was associated with an increased risk of autism spectrum disorder.

Recently, there has been a growth of literature on staff training and service adjustments for autism.

## Barriers to services encountered by people with autism

In a comprehensive review of literature about the barriers to services encountered by people with autism, Walsh et al^5^ highlighted the wide range of psychiatric comorbidities such as anxiety, depression and psychosis that they experience. These, along with medical conditions, can lead poorer health outcomes, lower quality of life and increased mortality among people with autism, compared with the general population. They noted that both children and adults with autism have a higher incidence of unmet healthcare needs compared with typically developing persons. Healthcare access is conceptualised as the fit between patient factors and system factors, encompassing the factors of availability, accessibility, accommodation, affordability and acceptability. Access includes the ability to identify healthcare needs, seek services, reach resources, obtain or use services and be offered services appropriate for needs. The authors outline the following four key themes within which reasonable adjustments could be considered: autism-related characteristics produced difficulties regarding crowded environments, loud noise, bright lights and suboptimal communication under stress, exacerbated by waiting; healthcare organisation issues highlighted poor autism knowledge and training among staff, leading to poor effectiveness, fear of stigma and rigid responses; system issues leading to poor guidelines, staff shortages and lack of support; and patient-related factors, such as attitudes toward autism and its management, as well as complex family relationships.

This framework allows for a systematic consideration of the corresponding reasonable adjustments that can be put in place to improve accessibility and effectiveness of services.

## What adjustments do people with autism want?

Adults with autism emphasised the importance and availability of key adjustments that could improve accessibility and acceptability of healthcare services. Brice et al^6^ recently reported that these were the sensory environment, the clinical and service context, and clinician knowledge and understanding. Preferred sensory environments included rooms with smaller numbers of people and lower levels of noise and light. Adjustments in the clinical services include increasing the length of appointments, offering remote consultations, reducing the number of appointments to be attended, providing clinical and historical information to the clinician to aid preparation and reduce repetition, using an easily identified and accessible location, reducing waiting times and providing support for the appointment itself. With regards to clinician knowledge and understanding, high importance was given to an understanding of autism, an easily identified and familiar clinician, and having an opportunity to ask questions about the conclusions reached.

Exploring the desired adjustments to psychotherapy in adults with autism, Lipinski et al highlighted the importance of consistent timings for appointments, structured sessions, the use of written information to complement the conversation, and the clinician's understanding of autism.^7^

## Skills and knowledge – a prerequisite for reasonable adjustments

The recently published ‘National Autism Strategy for England’^8^ gives priority to reducing health inequalities by ‘improving early identification, reducing diagnosis waiting times and improving diagnostic pathways for children and adults’. The Government in England has committed to introducing mandatory training on learning disabilities and on autism for health and social care staff.^9^ It is working with all professional bodies and devolved administrations to agree a common ‘core curriculum’, based on the recently published ‘Core Capabilities Framework for Supporting Autistic People’.^10^ Training requirements will, however, vary according to staff roles and interactions with people with autism, and will have to be adjusted accordingly because most people with autism with mental health difficulties need to access mainstream mental health services. Lack of autism-related training and skills across staf, this becoming disproportionate when comparing intellectual disability units with general mental health units particularly regarding working in these units (psychiatrists 94% skills in intellectual disability units *v*. 6% specialist skills in general mental health units).^11^

If people with autism are not to be disadvantaged when accessing mainstream mental health services, psychiatrists need to assess, diagnose and treat mental health problems in most people with autism. Psychiatrists need to face this challenge to reduce the risk of overmedication. The need to avoid overmedication has been highlighted for adults and children with autism.^12^ Onward specialist referral should only be considered in rare cases of complex comorbidity or diagnostic difficulty. Waiting times of 1–3 years for an assessment are frequent, depending on the geography and age of the person being assessed.^13^ Pasco^14^ summarises evidence that diagnosis in early childhood can be a gateway to specialist services, particularly educational. Benefits ae reported in social communication and core skills, as well as communication with parents, accompanied by reduction in stress and anxiety in parents and other family members. Without a significant change in psychiatric training and practice, the situation is unlikely to improve.

According to the framework that the Department of Health and Social Care in England uses for guidance, psychiatrists should be able to (a) implement reasonable adjustments when treating patients with autism to make the process as accessible and comfortable as possible; (b) identify the presence of autism, make a diagnosis where appropriate (cases that sufficiently straightforward as not to require a specialist autism opinion) and, when necessary, engage in the process of getting a wider assessment; (c) assess and treat the usual range of mental health disorders in patients with autism; (d) analyse why people with autism are at increased risk of mental health conditions; (e) understand the potential role of traumatic events in the lives of people with autism, their families and peers, such as childhood neglect and bullying, and be able to provide trauma-informed care so that people with autism are not further traumatised by services; (f) understand that people with autism commonly need adapted mental health therapies, and that certain therapies (such as group cognitive–behavioural therapy) are likely to be both ineffective if not adapted and very challenging to access because of sensory, language and social challenges; (g) develop, support and deliver community-based services to minimise the use of compulsion under the Mental Health Act.^10^

These core capabilities overlap significantly with the key competencies psychiatrists need when working with people with autism.

## Developing skills across mental health services

Staff in mental health services will need differing levels of skill and knowledge about autism to provide an adequate service for people with autism. Mental health professionals should be more autism-friendly to an undiagnosed patient if they suspect this approach is needed.

**Fig. 1:**
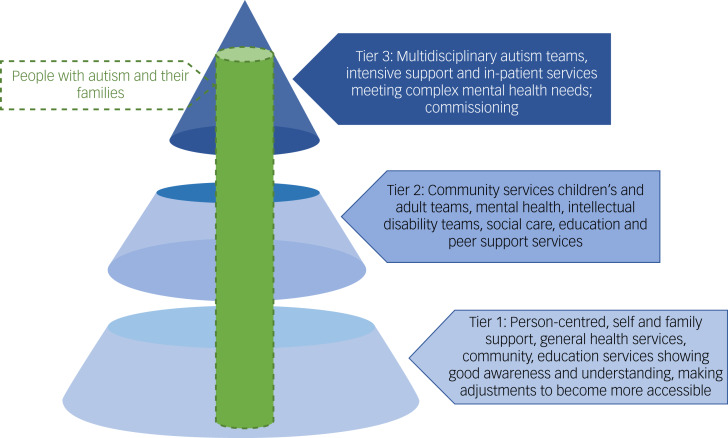
Autism skill tiers.

People with autism and their families may encounter various levels of service depending on need. Regarding the provision of person-centred services, a tiered approach to levels of competency would target training depending on individual roles within teams and services. Tier 3 would include staff in the full range of specialist autism teams, intensive support teams and in-patient services. Tier 2 would include staff in mental health and intellectual disability services across all age groups, as well as social care, education and other sectors dealing with people with autism. Tier 1 includes all staff in universal services accessed by the whole population. This is diagrammatically represented in Fig. 1.

Reasonably adjusted services provide the optimum environment for people with autism as they make their journey through life. This requires an appropriately trained, autism-friendly workforce ensuring provision of timely, person-centred support.

## Key messages

Provision of mental healthcare to people with autism is a high priority. Current mental health services are unable to meet the rising demand, leading to delays in identification, support and treatment. There are well-recognised barriers to services and lack of agreement of the reasonable adjustments needed to overcome them. Staff in mainstream mental health services have insufficient training and skills in this area, and therefore have difficulty adapting services to accommodate autism-related needs of patients irrespective of diagnosis. The autism capability framework provides an opportunity to develop standardised high-quality training for all staff, including psychiatrists. It would enable psychiatrists to be at the forefront of workforce transformation, and develop the services that people with autism so urgently need.

## References

[1] World Health Organization (WHO). *ICD-11 Mortality and Morbidity Statistics*. WHO, 2020 (https://icd.who.int/browse11/l-m/en).

[2] Berney T, Bevan R, Brugha T, Carpenter P, Clarke F, Doherty M, *The Psychiatric Management of Autism in Adults (College Report 228).* Royal College of Psychiatrists, 2020 (https://www.rcpsych.ac.uk/docs/default-source/improving-care/better-mh-policy/college-reports/college-report-cr228.pdf?sfvrsn=c64e10e3_2).

[3] Roman-Urrestarazu A, van Kessel R, Allison C, Fiona E, Matthews FE, Brayne C, Association of race/ethnicity and social disadvantage with autism prevalence in 7 million school children in England. JAMA Pediatr 2021; 175(6): e210054. 33779707 10.1001/jamapediatrics.2021.0054 PMC8008434

[4] Rai D, Lewis G, Lundberg M, Dalman C, Carpenter P, Magnusson C. Parental socioeconomic status and risk of offspring autism spectrum disorders in Swedish population-based study. J Am Acad Child Adolesc Psychiatry 2012; 51(5): 467–76.e6. 22525953 10.1016/j.jaac.2012.02.012

[5] Walsh C, Lydon S, O'Dowd E, O'Connor P: Barriers to healthcare for persons with autism: a systematic review of the literature and development of a taxonomy, developmental neurorehabilitation. Dev Neurorehabil 2020; 23(7): 413–30. 10.1080/17518423.2020.1716868 36112897

[6] Brice S, Rodgers J, Ingham B, Mason D, Wilson C, Freeston M, The importance and availability of adjustments to improve access for autistic adults who need mental and physical healthcare: findings from UK surveys. BMJ Open 2021; 11: e043336. 10.1136/bmjopen-2020-043336 PMC7978247 33737429

[7] Lipinski S, Blanke ES, Suenkel U, Dziobek I. Outpatient psychotherapy for adults with high-functioning autism spectrum condition: utilization, treatment satisfaction, and preferred modifications. J Autism Dev Disord 2019; 49: 1154–68. 30415320 10.1007/s10803-018-3797-1

[8] Department of Health and Social Care. *National Strategy for Autistic Children, Young People and Adults: 2021 to 2026*. Department of Health and Social Care, 2021 (https://www.gov.uk/government/publications/national-strategy-for-autistic-children-young-people-and-adults-2021-to-2026).

[9] Parkin E, Long R, Powell A, Jarrett T. *Autism – Overview of Policy and Services*. House of Commons Library Briefing Paper Number 7172. House of Commons Library, 2020 (https://researchbriefings.files.parliament.uk/documents/CBP-7172/CBP-7172.pdf).

[10] Skills for Health. *Core Capabilities Framework for Supporting Autistic People*. Skills for Health, 2019 (https://skillsforhealth.org.uk/info-hub/learning-disability-and-autism-frameworks-2019/).

[11] Jones K, Gangadharan S K, Brigham P, Smith E, Shankar R. Current practice and adaptations being made for autistic people admitted to inpatient psychiatric services across the UK. BJPsych Open 2021; 7(3): e102. 33988120 10.1192/bjo.2021.58 PMC8161595

[12] NHS England. *Stopping Over Medication of People with a Learning Disability, Autism or Both (STOMP).* NHS England, 2016 (www.england.nhs.uk/learning-disabilities/improving-health/stomp/).

[13] NHS Digital. Autism Statistics Quarter 1 (April to June) 2019-20 to Quarter 4 (January to March) 2020-21. NHS Digital, 2021 (https://digital.nhs.uk/pubs/autismsep21).

[14] Pasco G. The value of early intervention for children with autism. Paediatr Child Health 2018; 28(8): 364–7.

